# Microstructure and Corrosion of Mg-Based Composites Produced from Custom-Made Powders of AZ31 and Ti6Al4V via Pulse Plasma Sintering

**DOI:** 10.3390/ma17071602

**Published:** 2024-03-31

**Authors:** Anna Dobkowska, Mirosław Jakub Kruszewski, Jakub Ciftci, Bartosz Morończyk, Izabela Zgłobicka, Rafał Zybała, Łukasz Żrodowski

**Affiliations:** 1 Faculty of Materials Science and Engineering, Warsaw University of Technology, 141 Woloska St., 02-507 Warsaw, Poland; miroslaw.kruszewski@pw.edu.pl (M.J.K.); or jakub.ciftci@amazemet.com (J.C.); or bartosz.moronczyk@amazemet.com (B.M.); 2 Faculty of Mechanical Engineering, Bialystok University of Technology, Wiejska 45C, 15-351 Bialystok, Poland; i.zglobicka@pb.edu.pl; 3 Łukasiewicz Research Network, Institute of Microelectronics and Photonics, Al. Lotnikow 32/46, 02-668 Warsaw, Poland; 4 AMAZEMET Sp. z o. o. [Ltd.], Al. Jana Pawła II 27, 00-867 Warsaw, Poland; lukasz.zrodowski@amazemet.com

**Keywords:** metal matrix composite, AZ31 alloy, Ti6Al4V powder, metallic reinforcement, microstructure, corrosion

## Abstract

Magnesium (Mg) and its alloys offer promise for aerospace, railway, and 3D technology applications, yet their inherent limitations, including inadequate strength, pose challenges. Magnesium matrix composites, particularly with metallic reinforcements like titanium (Ti) and its alloys, present a viable solution. Therefore, this study investigates the impact of Ti6Al4V reinforcement on AZ31 magnesium alloy composites produced using pulse plasma sintering (PPS). Results show enhanced microhardness of the materials due to improved densification and microstructural refinement. However, Ti6Al4V addition decreased corrosion resistance, leading to strong microgalvanic corrosion and substrate dissolution. Understanding these effects is crucial for designing Mg-based materials for industries like petrochemicals, where degradation-resistant materials are vital for high-pressure environments. This research provides valuable insights into developing Mg-Ti6Al4V composites with tailored properties for diverse industrial applications, highlighting the importance of considering corrosion behavior in material design. Further investigation is warranted to establish predictive correlations between Ti6Al4V content and corrosion rate for optimizing composite performance.

## 1. Introduction

Magnesium and alloys are considered to be the most promising materials for aerospace [1], railway [2], as well as 3D (computer, communication and consumer electronic) applications [3]. However, taking into account properties of Mg and alloys, insufficient strength, stiffness, and modulus, it may be difficult to meet requirements of these applications, especially for those working at high temperatures [4,5]. Magnesium matrix composites may be manufactured using solid process, infiltration, or powder metallurgy methods [6]. Selection of the proper technique is key because of the high reactivity of Mg, which may lead to significant problems within the manufacturing process.

One of the most common obstacles that limits wide application of Mg and Mg-based alloys is unsatisfactory mechanical properties. According to Zhou et al. [7], the mechanical properties of Mg and Mg-based alloys may be improved via grain refinement (mainly deformation methods) or by adding reinforcement. Mg-based metallic matrix composites (MMCs) have become very attractive, and they were produced using various reinforcement techniques. Bochenek et al. [8] stated that reinforcement should be in the form of hard and stiff particles. Dinharan et al. [9] observed that reinforcement in the form of ceramic particles causes major loss in the ductility of Mg matrix composites. Thus, they proved that particles that possess a higher melting point should be chosen. Furthermore, not only the type of particles, but also volume fraction, homogeneous distribution in the matrix, as well as interaction between the matrix and strengthening particles, have an impact on the properties of the Mg-based composites.

The most extensively used are SiC particles, which are characterized by high strength, high stiffness, and stable chemical activity. According to [6,10], SiC reduce the ductility of Mg while increasing yield strength and elastic modulus. Such behavior unfavorably affects the use of composites in structural applications. Chua et al. [6] used SiC particles as a reinforcement in AZ91-based MMCs. The authors investigated the influence of the particle size and thermal shock on the mechanical properties of composites, arising from the effect of the reaction between the matrix and the filler. The results allowed us to observe two phases, major Mg_17_Al_12_ and secondary Mg_2_Si. As stated, the mechanical properties of the composites are affected by the Mg_2_Si phase, whose presence favors embrittlement.

The increased attention on metal particles over ceramics, i.e., Al, Zn, Cu, Ti, Sb, Ca, Bi, Pb, Mn, and rare-earth (RE) metals [11,12,13,14,15,16,17,18,19,20], is caused by their excellent mechanical properties, improved corrosion resistance, as well as a remarkable strengthening and toughening effect on Mg and Mg alloys [6,21]. Because of the unfavorable behavior of SiC particles, metallic particles with higher strength that are similar to Mg’s physical characteristics are more promising. There were investigations about the reinforcing effect of Cu [22], pure Ti [23,24], and Ti alloys [25]. The combination of Ti and Mg seems to be the most appropriate because of the hexagonal crystal structure (hcp) of both metals, which would alleviate compatibility problems. In addition, Ti exhibits mechanical deformation that surpasses that of ceramic particles. This may help to enhance ductility. Ti and Mg do not mix, either in solid or liquid state [26,27]. Severe plastic deformation allows to develop metastable phases [26]. Manufacturing methods of MMCs with reinforcement in the form of Ti particles are: disintegrated melt deposition [24], powder metallurgy [23,28], accumulative diffusion bonding [29], stir casting [30], vacuum hot pressing [15], and mechanical alloying [31]. These techniques allow to successfully reinforce pure Mg as well as Mg alloys, such as AZ31, AZ61, AZ91, AM50, and AME505. Nevertheless, some of the unwanted microstructural features have been reported, i.e., improper distribution [24], clustering of particles and pores at macro and micro levels [23], weak interface [28], coarse grains [28], emergence of detrimental compounds [30,31], high energy and time consumption [29,31], and distortion of crystal structure [27]. These features affect the properties of composites but also increase the cost of production. In recent years, many Mg alloys and Mg composites were developed to possess controlled but fast degradation rates and enhanced mechanical properties. This combination allows for their wide use in the oil industry, where high corrosion rates and high mechanical properties are the advantages of the fracturing temporary plugging tools in oil and gas exploitation [32].

Although there are Mg alloys with high mechanical properties, their values are still not sufficient. Therefore, to fasten corrosion rates, some reinforcement may be used. Magnesium alloy (AZ31) matrix composites reinforced with Ti particles was prepared via friction stir processing (FSP) by Dinharan et al. [9]. Authors investigated microstructure and tensile behavior of the fabricated composites. Manufacturing methods allow to obtain uniformly distributed filler, which did not react with matrix and did not decompose. Furthermore, Ti particles were also resistant to severe plastic strain without breakage. According to Candan et al. [33] and Ai et al. [34], addition of Ti (0.2–0.8 wt. %) leads to strengthening at room temperature as well as improvements in corrosion resistance of the cast AZ91 alloy. Even smaller amounts of Ti reinforcement (0.01–0.02 wt. %) drastically improve deformation behavior of Mg-6Al-1Zn alloy at high temperatures. Xi et al. [25] reinforced magnesium (ZK51 alloy) matrix composite via powder metallurgy routes using Ti6Al4V (TAp) particles. The findings indicate that the tensile strength and elastic modulus of produced composites are greater than those of the unreinforced magnesium alloy. Furthermore, TAp seemed to be more favorable for ductility when compared to composites reinforced with SiC particles. One of the effective methods for preparation of AZ31-Ti composites is mechanical milling. Zhou et al. [7] observed that increases in time of the milling process result in decreases in size of Mg matrix crystallite. Such a process caused homogeneity in composite. The repeated deformation, as well as welding and fracture mechanisms, have been noted. In terms of obtained properties, with the highest Ti addition, the microhardness also increased. The mechanically milled AZ31-Ti particles exhibited larger yield strength, both at room and at elevated temperature (300 °C).

Since most research focused only on pure Ti addition as a composite filler, filler in the form of Ti6Al4V needs to be investigated in terms of structural and corrosion properties of Mg-based composites. Therefore, the aim of this study was to investigate the addition of 10, 20, and 30 vol.% of Ti6Al4V particles on the properties of AZ31 + Ti6Al4V composite produced by plasma-assisted methods, particularly pulse plasma sintering (PPS).

## 2. Materials and Methods

### 2.1. Materials

**Figure 1:**
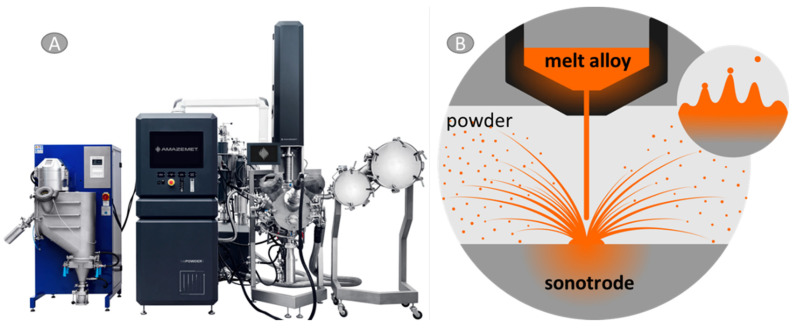
AZ31 powdering: (**A**) rePowder device by Amazemet, (**B**) schematic of powdering.

For the needs of this work, mixtures composed of custom-made AZ31 powder with Ti6Al4V addition (Ti6Al4V Grade 5, TLS Technik GmbH, Bitterfeld, Germany) were prepared. AZ31 in the form of rods was atomized by ultrasonic atomization with rePowder device, AMAZEMET Sp. z o. o, Warsaw, Poland (Figure 1A). Atomization was carried out with induction melting in a graphite crucible at 950 °C. After the melting step material had been released onto a refractory sonotrode plate, the pouring rate was controlled by a differential pressure of ~0.15 mbar between the induction furnace and atomization chamber plate vibrating at the frequency of 40 kHz, where the atomization took place (Figure 1B). The relative amplitude of vibrations was set at 75% during the processes. Atomization took place in the vacuum chamber with Ar gas overpressure. The oxygen level during the atomization was kept below 50 ppm.

Powders of AZ31 and Ti6Al4V were weighted to obtain powder mixtures of AZ31+ x vol.% Ti6Al4V, where x = 10, 20, and 30. Then, the powders were mixed for 2 h in a turbula-type mixer to form homogeneous mixtures for the PPS compaction. No milling balls were used to avoid any contamination.

For the PPS consolidation, the powders were placed in a cylindrical graphite die located between two graphite punches, and the initial uniaxial compressive pressure of 25 MPa was applied. Afterwards, powders were heated up to 400 °C with a heating rate of 90–100 °C × min^−1^, after which the final pressure of 60 MPa was applied. The sintering temperature was maintained for 5 min. The whole PPS process was conducted in vacuum (5 × 10^−3^ Pa). As a result, samples with 10 mm diameter and c.a. 10 mm in height were produced. Pure AZ31 alloy was also sintered as the reference. The experimental density (bulk density) of the fabricated materials was measured by the Archimedes method. The theoretical density was calculated using the rule of mixture. Bulk density was calculated using Equation (1):

(1) $$\rho_{B}=\frac{m_{d}}{m_{sat}-m_{sus}}\cdot \rho_{H_{2}O}$$

where *ρ_B_*—the bulk density (g/cm^3^), *m_sat_*—saturated mass (g), *m_d_*—dry mass (g), *m_sus_*—suspended immersion mass (g), and $\rho_{H_{2}O}$—density of water at RT.

Details about PPS can be found in [35].

### 2.2. Methods

Particles size distribution (PSD) of the powder mixtures were examined using a laser particle size analyzer (Horiba LA950, Horiba, Warsaw, Poland) in an isopropanol suspension. Parameters such as d10, d50, d90, and average size of the particles were measured. The powder mixtures were observed using a scanning electron microscope (SEM, Hitachi SU 8000, Japan) equipped with an energy-dispersive spectroscopy (EDX) detector.

The microstructure of the materials was described based on SEM observations in backscatter mode (BSE). Electron backscattered diffraction (EBSD) was utilized using a scanning electron microscope (SEM, Hitachi SU70, Hitachi Ltd., Tokyo, Japan) equipped with a Bruker EBSD detector. Samples were ion-milled in an Ar beam (Ion Milling System, Hitachi IM4000, Hitachi Ltd., Tokyo, Japan). All EBSD measurements were conducted with a step size of 0.2 µm. The crystallographic orientation of grains is presented in the form of inverse pole figures (IPF).

Electrochemical testing of the sintered materials was performed in naturally aerated 0.1 M NaCl at room temperature. The Gamry Ref 600+ potentiostat was used. Measurements were performed in a Faraday cage in a large-volume electrochemical cell (500 mL). A three-electrode setup was used, where Pt was a counter electrode, Ag/AgCl was a reference electrode, and a tested material was a working electrode. Measurements were composed of an open circuit potential (E_OCP_) registration during 1 h of immersion, and potentiodynamic polarization was performed. Potentiodynamic scans were registered starting from 0.5 V below E_OCP_ and finishing at 2 V vs. E_Ref_ at scan rate of 5 mV/s. To have further insight into the corrosion processes occurring on the surfaces of the investigated materials, SEM observations of corrosion morphology after 1 h of immersion in 0.1 M NaCl were performed (SEM Hitachi SU 8000, Hitachi Ltd., Tokyo, Japan). Both surfaces with and without corrosion products were observed. Corrosion products were removed by chemical treatment with CrO_3_ for 40 s as specified in Pałgan et al. [36]. Additionally, the corrosion rate of the materials was calculated using the hydrogen release method, as previously described in [37]. To ensure the reproducibility of results, at least three specimens from each composition were tested.

Vickers microhardness testing was conducted under a load of 200 g using Innovatest Falcon 500 Micro/Macro Vickers Tester (Innovatest GmbH, Selfkant, Germany); 10 points were measured on each material.

## 3. Results

### 3.1. Precursor Characterization

**Figure 2:**
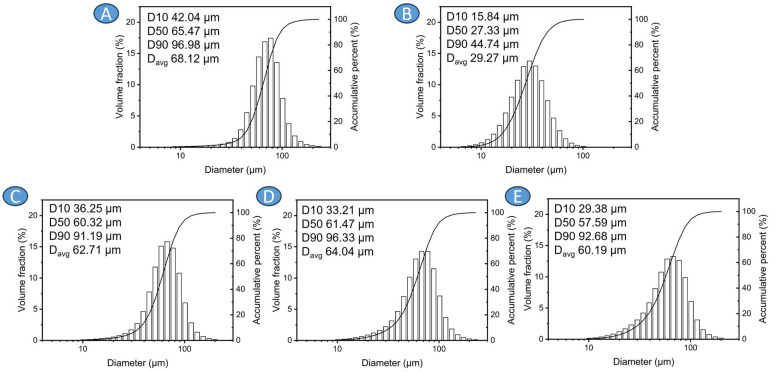
Particle size distributions shown together with d10, d50, d90, and average particles size for precursors and powder mixtures: (**A**) AZ31, (**B**) Ti6Al4V, (**C**) AZ31 + 10 vol.% Ti6Al4V, (**D**) AZ31 + 20 vol.% Ti6Al4V, (**E**) AZ31 + 30 vol.% Ti6Al4V.

**Figure 3:**
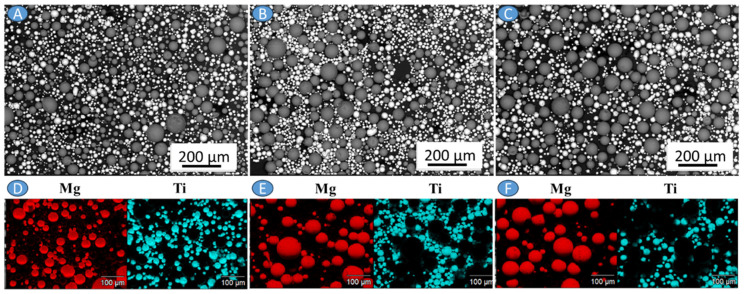
SEM imaging of the powder mixtures: (**A**) AZ31 + 10 vol.% Ti6Al4V, (**B**) AZ31 + 20 vol.% Ti6Al4V, (**C**) AZ31 + 30 vol.% Ti6Al4V with corresponding mapping of Mg and Ti in the powders (**D**–**F**).

The AZ31 powder (Figure 2A) is characterized by larger particles than Ti6Al4V powders (Figure 2B). The main reason for this difference is the powder production method, which for AZ31 powder involves ultrasonic atomization, while Ti6Al4V powders were manufactured by gas atomization. This is especially visible in Figure 3, where SEM imaging with corresponding EDX analyses for Mg and Ti are made. Almost all of the tested reinforcing powder particles were below the d50 parameter of the AZ31 powder, which forms the matrix of the mixtures. Precursors exhibit an average size greater than the d50 parameter (median) of the particle size distribution, causing histograms characterized by right-skewed distribution. This is also noticeable for particle size distributions obtained for mixtures, especially ones containing 30% reinforcing powder, due to the increase in fine particles resulting from the addition of Ti6Al4V, for which the d90 value is 44.74 µm (Figure 2C–E).

### 3.2. Materials Characterization

**Figure 4:**
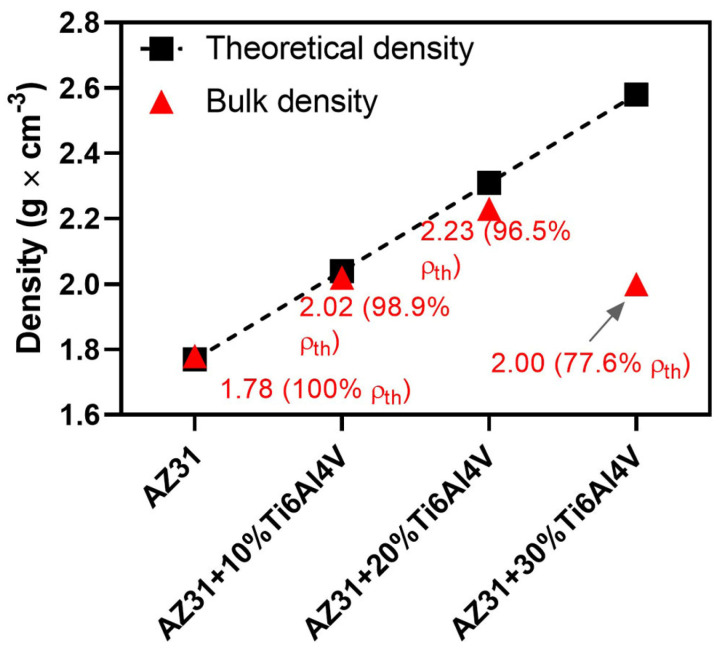
Density of the sintered materials.

The densities of the fabricated pure AZ31 alloy and AZ31-based composites are presented in Figure 4. After the consolidation at 400 °C for 5 min, the reference material was characterized by full densification. As the amount of reinforcement increases, one can observe that the densification starts to drop. For AZ31-Ti6Al4V composites, we achieved a visible drop in densification: 98.9, 96.5, and 77.6% for 10, 20, and 30 vol.% of Ti6Al4V, respectively.

**Figure 5:**
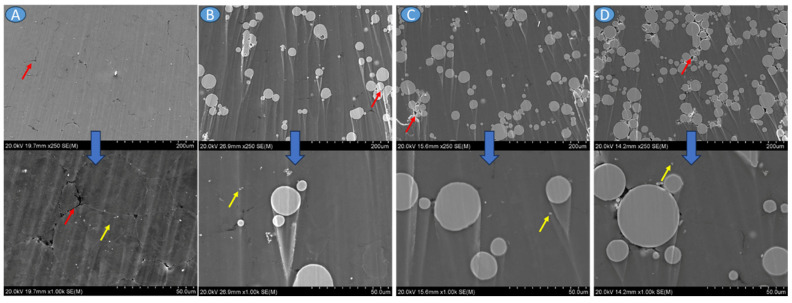
SEM-BSE images of the (**A**) AZ31, (**B**) AZ31 + 10 vol.% Ti6Al4V, (**C**) AZ31 + 20 vol.% Ti6Al4V, and (**D**) AZ31 + 30 vol.% Ti6Al4V; red arrows show voids, yellow arrow—small precipitations.

**Figure 6:**
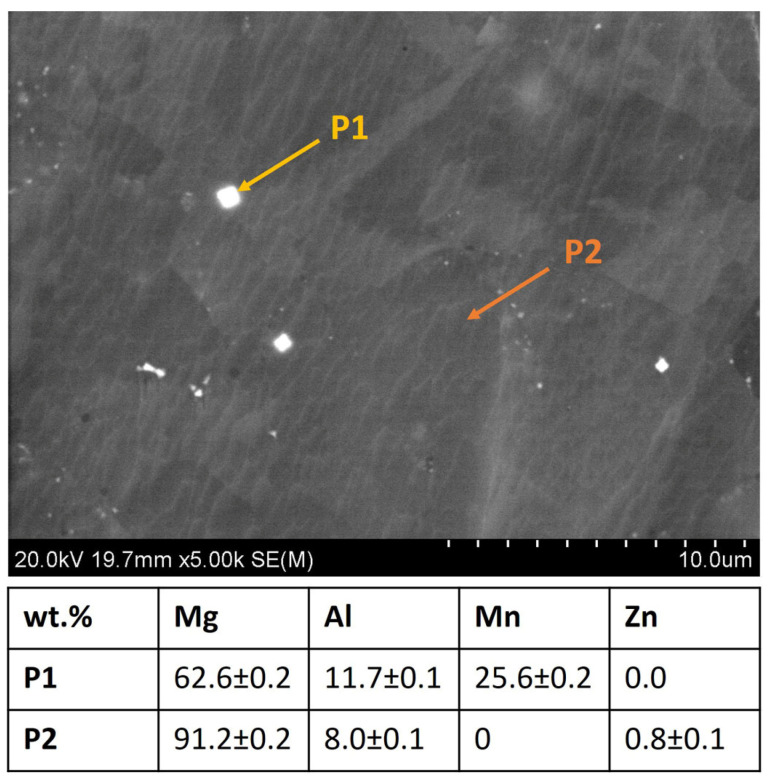
EDX analysis of the precipitations marked as P1 and Mg matrix marked as P2, performed in the AZ31 alloy.

**Figure 7:**
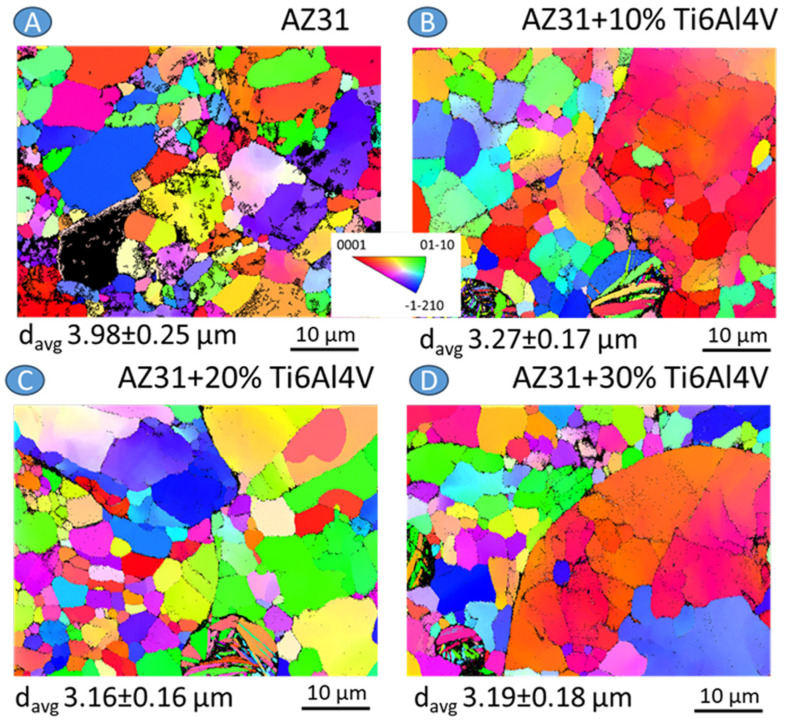
EBSD results in the form of inverse pole figure (IPF) maps presented for the investigated materials: (**A**) AZ31, (**B**) AZ31 + 10 vol.% Ti6Al4V, (**C**) AZ31 + 20 vol.% Ti6Al4V, and (**D**) AZ31 + 30 vol.% Ti6Al4V.

Analyzing SEM-BSE images of the investigated materials shown in Figure 5, it can be noted that the Ti6Al4V particles adhered well to the Mg matrix; no pores or voids in the particle–matrix interfaces were observed. Some randomly distributed pores were observed for all materials. Considering AZ31, pores were formed in the triple points of particle interfaces (Figure 5A). In the case of composites mostly located between Ti6Al4V, particles are observed and marked by the red arrows (Figure 5B–D). The small white precipitates were formed in AZ31 alloy, and they were also present in the AZ31 + Ti6Al4V composites (yellow arrows in Figure 5). Those white precipitates were enriched in Al and Mn (Figure 6). All investigated materials have randomly oriented grains (Figure 7), with the smaller grain size observed for the composites.

**Figure 8:**
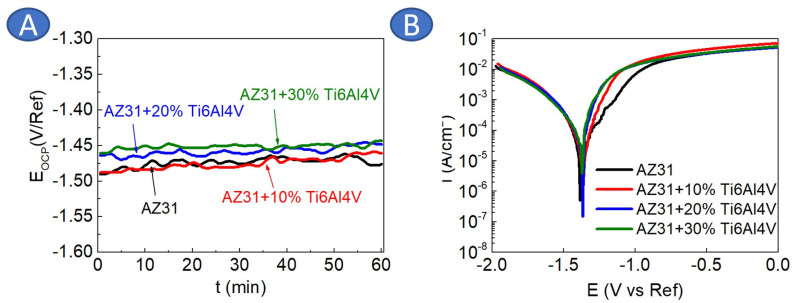
Results obtained from the electrochemical testing in 0.1 M NaCl for AZ31 and AZ31 with Ti6Al4V addition: (**A**) open circuit potential evaluation, (**B**) potentiodynamic curves.

The potential recorded under open circuit conditions is presented in Figure 8A. As observed, the E_OCP_ recorded for the AZ31 and AZ31 + 10% Ti6Al4V are oscillating around the same values (−1.48 V/Ref). The addition of 20 and 30 vol.% of Ti6Al4V shifted EOCP towards more positive values, reaching −1.45 V/Ref for the AZ31 + 30 vol.% Ti6Al4V. All potentiodynamic curves are located in the same range of current densities, and only slight shifts are observed (Figure 8B). Nevertheless, the addition of Ti6Al4V to AZ31 changed the corrosion mechanism from passivation characteristics for AZ31 to active dissolution for the composites. The characteristic inflection point is observed on the anodic part of the potentiodynamic curve recorded for AZ31, and it can be identified with breakdown potential (E_b_ = −1.26 V/Ref). The average values obtained from Tafel extrapolation are given in Table 1. As observed, the addition of 10 vol.% Ti6Al4V did not increase values of corrosion current densities (i_corr_) significantly. There is a distinct difference between corrosion current densities calculated for higher concentrations of Ti6Al4V (i.e., 20 vol.% and 30 vol.%) than for AZ31 and AZ31 with 10 vol.% Ti6Al4V.

**Figure 9:**
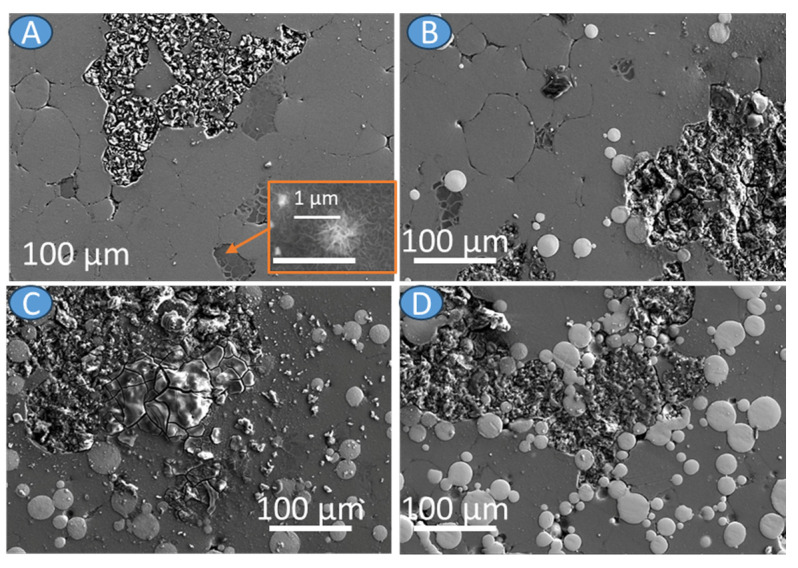
Characterization of corroded surfaces with corrosion products (**A**) AZ31, (**B**) AZ31 + 10 vol.% Ti6Al4V, (**C**) AZ31 + 20 vol.% Ti6Al4V, (**D**) AZ31 + 30 vol.% Ti6Al4V.

**Figure 10:**
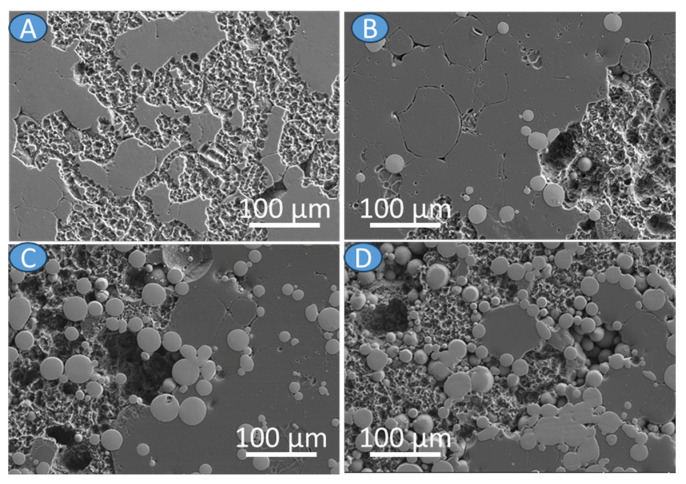
Characterization of corroded surfaces after chemical removal of corrosion products (**A**) AZ31, (**B**) AZ31 + 10 vol.% Ti6Al4V, (**C**) AZ31 + 20 vol.% Ti6Al4V, (**D**) AZ31 + 30 vol.% Ti6Al4V.

To have better insight into corrosion performance of the investigated materials, the corrosion rate was calculated on the total hydrogen release during immersion in the solution, and the results are given in Table 2. The lowest corrosion rate was calculated for the AZ31, and with the increasing Ti6Al4V addition, the corrosion rate increased; however, no linear trend is observed. The addition of 30 vol.% of Ti6Al4V increased corrosion rate more than two times when compared to the AZ31. The presented data are in agreement with the SEM observations (Figure 9 and Figure 10). In all cases, the microgalvanic mechanism is observed, with the AZ31 being the anodic region and Ti6Al4V serving as the cathode. The corrosion products formed on the surfaces loosely adhered to the bulk materials (Figure 9). When corrosion products were removed, the differences in the corrosion propagation are clearly visible. The corrosion on the AZ31 was propagating on the surface of the alloy along some privileged paths with some deeper pits (Figure 10A), while strong dissolution of the AZ31 substrate was observed in the case of AZ31 + Ti6Al4V (Figure 10B–D). In those cases, as a result of strong microgalvanic corrosion between AZ31 and Ti6Al4V, corrosion damage formed into the depth of the substrate.

**Figure 11:**
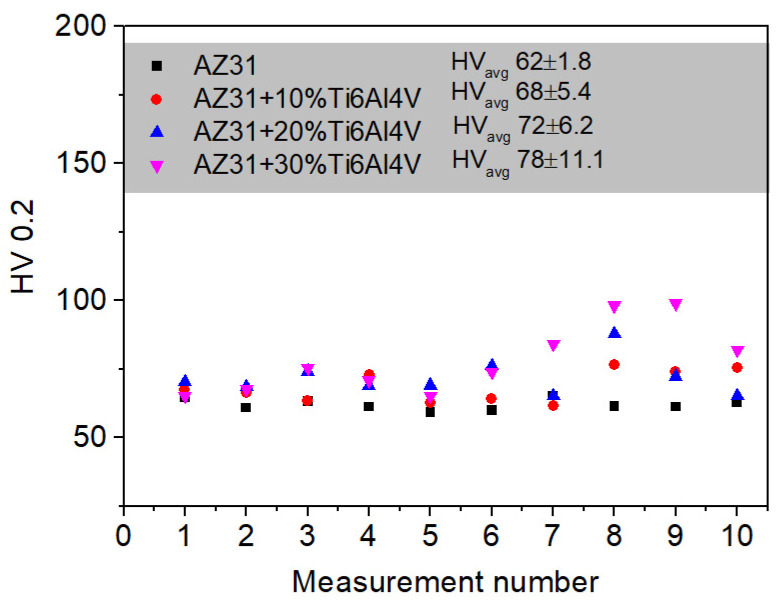
Microhardness of the investigated alloys.

Microhardness of the investigated materials was determined to provide insight into the effect of Ti6Al4V particle addition on the hardness of the AZ31 matrix (Figure 11). As observed, the microhardness of the matrix increased together with the Ti6Al4V addition, and the average values were found to be around 62 ± 1.8, 68 ± 5.4, 72 ± 6.2, and 78 ± 11.1 HV0.2 for AZ31, AZ31 + 10 vol.% Ti6Al4V, AZ31 + 20 vol.% Ti6Al4V, and AZ31+ vol. 30% Ti6Al4V, respectively. Ti6Al4V addition increased microhardness; however, an increase in standard deviation is significant. This may be related to dispersion inhomogeneities of the reinforcing phase, which is a common occurrence in MMC. We observed agglomeration of the reinforcement at the highest concentration of the particles (Figure 5), which confirms our hypothesis.

## 4. Discussion

In this study, custom-made AZ31 powder with various additions of Ti6Al4V (10, 20 and 30 vol.%) were sintered in the form of cylinders. Materials were produced at 400 °C, which is a relatively low compaction temperature compared to other reported studies for this type of alloy. Paraskevas et al. [38] conducted SPS consolidation of recycled AZ31 chips above 470 °C to achieve increased flow behavior and thus high densification. Yang et al. [39] reached almost theoretical density with a hot-pressing device where the consolidation temperature ≥ 535 °C. Muhammad et al. [40] fabricated, by SPS, almost fully densified AZ31 materials starting from 450 °C. Nevertheless, sintering under the parameters used in this study achieved full densification of AZ31 and almost full densification of AZ31 + Ti6Al4V, as the results show. The densities of the AZ31 + Ti6Al4V were higher than the density of composites reinforced with SiC [40]. Although the size of Ti6Al4V was bigger than AZ31, the particles adhered well to the Mg matrix and did not change their size or morphology. Since agglomerates of Ti6Al4V particles were observed in the materials, it is worth considering the use of grinding media for the mixture preparation process in the future.

The microstructure formation in the case of AZ31 and AZ31 with the addition of Ti6Al4V was slightly different, as Ti6Al4V caused smaller grain size formation in the matrix of the composites. The addition of Ti6Al4V completely changed the corrosion mechanism of AZ31. The homogeneously distributed corrosion paths were found to be formed on the surface of AZ31. The addition of Ti6Al4V caused a strong microgalvanic effect and the dissolution of the AZ31 matrix. Ti particles have been previously found to possess a more noble potential compared to that of AZ31 alloy [41]. Corrosion propagated into the depth of the material, causing significant damage to the substrate. Still, the major anodic reaction was Mg dissolution. The observed mechanism is stronger than the corrosion damage observed for Mg reinforced with ceramic particles produced using the same technique [42]. One can notice that the agglomeration of Ti6Al4V particles intensified corrosion propagation, in agreement with the rule that a bigger cathodic area caused faster dissolution of anodic areas, and this phenomenon was not compensated by smaller grain size formed in the composites. Similar behavior was observed by Jiao et al. [41]; however, they noticed much higher corrosion rates for similar types of composite due to more tests performed in more concentrated chloride solutions. Our findings may be useful for the petrochemical industry, where Mg-based materials are known as matrix for degradable fracturing tools needed in the oil and gas extraction processes where high pressure is also present. Nevertheless, we were not able to obtain a linear relationship between Ti6Al4V content and corrosion rate of the investigated materials, which would support prediction of tools lifetime under operating conditions.

## 5. Conclusions

This study highlights the potential of Ti6Al4V reinforcement in enhancing the mechanical properties of AZ31 magnesium alloy composites produced via pulse plasma sintering. The incorporation of Ti6Al4V leads to improved densification and microstructural refinement, contributing to enhanced mechanical properties. However, the addition of Ti6Al4V alters the corrosion mechanism of the composites, resulting in microgalvanic corrosion and substrate dissolution. This finding underscores the importance of considering corrosion behavior in the design of Mg-based materials for industrial applications, particularly in environments with chloride exposure. Further research is needed to establish a predictive relationship between Ti6Al4V content and corrosion rate to optimize the performance and lifespan of AZ31 + Ti6Al4V composites in practical applications. Overall, the study provides valuable insights into the development of Mg-based materials with tailored mechanical and corrosion properties for diverse industrial applications, including aerospace, transportation, and petrochemicals. These conclusions highlight the potential of Mg + Ti6Al4V composites and emphasize the need for further research to optimize composite performance and lifespan in practical applications.

## Figures and Tables

**Table 1:** Electrochemical parameters extrapolated using Tafel method for the investigated materials (i_corr_—corrosion current density, E_corr_—corrosion potential).

Material	i_corr_ (µA/cm^2^)	E_corr_ (V vs. Ref)
AZ31	10 ± 2	−1.38 ± 0.1
AZ31 + 10 vol.%Ti6Al4V	8 ± 2	−1.37 ± 0.1
AZ31 + 20 vol.%Ti6Al4V	33 ± 7	−1.37 ± 0.1
AZ31 + 30 vol.%Ti6Al4V	28 ± 11	−1.37 ± 0.1

**Table 2:** Corrosion rate calculated based on hydrogen release method (CR-corrosion rate).

Material	CR (mpy)
AZ31	6.2 ± 0.2
AZ31 + 10 vol.% Ti6Al4V	6.8 ± 0.1
AZ31 + 20 vol.% Ti6Al4V	8.7 ± 0.2
AZ31 + 30 vol.% Ti6Al4V	14.5 ± 0.3

## Data Availability

Data are contained within the article.
